# Organoids at the forefront of global health: accelerated research and ethical implications in the cases of Zika and COVID-19

**DOI:** 10.1080/17441692.2025.2496679

**Published:** 2025-05-06

**Authors:** Amy Hinterberger, Aleksandra Stelmach

**Affiliations:** a Department of Bioethics and Humanities, University of Washington, Seattle, WA, USA; b Department of Global Health and Social Medicine, King's College London, London, UK

**Keywords:** Organoids, global health, ethics

## Abstract

Bioengineering technologies are increasingly important in global health research, yet their applications beyond vaccines and diagnostics remain underexplored. Our paper examines the role of organoids – advanced stem cell technologies used to model human organs, such as lungs and brains – in the context of infectious disease research. Organoids became crucial during the Zika and COVID-19 outbreaks. These new model systems enabled rapid insights into pathogen behaviour. We analyse how the urgency of Zika and Covid-19 accelerated organoid research, tracing its rise and subsequent slowdown. Our investigation reveals that while organoid technologies experienced a burst of activity during these emergencies, their momentum has waned, with ongoing research predominantly focusing on diseases prevalent in the Global North. We argue that the uneven acceleration and subsequent deceleration of organoid research underscores a critical need for equitable integration of bioengineering in global health priorities, particularly in the context of pandemic preparedness. Our findings advocate for a balanced and inclusive strategy to enhance pandemic preparedness and address global health disparities effectively.

## Introduction

Emerging technologies such as bioengineering have long been viewed as holding tremendous promise for addressing global health challenges. Yet, as Piot (2012) argued over a decade ago, this potential was not realised, ‘with a notable exception of vaccines’ (p. 46). This has changed since the Covid-19 pandemic which prompted a renewed interest in bioengineering research for global health (Bowden et al., 2023). Here we explore organoid technologies, a cutting-edge bioengineering technique which played a key part in the pandemic response to Covid-19, and in the response to the 2015–2016 Zika virus outbreak in the Americas. As organoids provide a unique platform for experimental virology (Sanyal & Paul, 2021), we explore the social and ethical issues arising for organoid research in the context of global health.

Organoids are miniature versions of organs and tissues grown often from induced pluripotent stem cells (iPSCs). The iPS cells are derived from somatic adult cells, such as skin cells. They are reprogrammed back to their embryonic stem cell-like state and then grown into cells of a specific organ. When put in the dish and in right conditions, these cells self-organise and mimic the structure and functions of the brain, lungs, kidney, and other human organs. Such three-dimensional structures provide better models than traditional two-dimensional cultures of how cells behave in a living organism, and organoids derived from huma stem cells are considered a better proxy of the human body than organoids derived from animal cells. The emergent properties of organoids have opened new horizons for research, offering unprecedented insights into the studies of development, drug screening and modelling disease (Corsini & Knoblich, 2022).

The global health emergencies of the Zika virus outbreak and the Covid-19 pandemic pushed this field into the frontiers of studies of infectious disease and emerging pathogens. The sudden acceleration, and even ‘explosion’ of organoid research (Yan et al., 2024) that ensued helped establish how emerging pathogens behave, how they affect human body and which treatments might work. These aspects were key to a rapid outbreak response. The rise of organoid research for infectious diseases has been held up as a key example of how research and development can be accelerated and mobilised to support outbreak responses, pandemic preparedness and the global health research agenda more broadly (Blutt & Estes, 2022; Sanyal & Paul, 2021).

The acceleration of R&D during the Covid-19 pandemic resulted in a development of new biotechnological products at a record speed and renewed the interest of governments, funders, and various institutions in the potential of biotechnology for infectious disease research (e.g. UK Government, 2023). Many of these discussions have so far centred on vaccines, diagnostic tools and therapeutics. By contrast, little attention has been paid to technologies developed for basic research, and the role they play in health emergencies has not been investigated (but see for example Long, 2023). We address this gap by considering the implications of organoid technologies for global health and pandemic preparedness.

In this article, we argue that the uneven acceleration and subsequent deceleration of organoid research underscores a critical need for equitable integration of biotechnologies in global health priorities, particularly in the context of pandemic preparedness. To make this argument we interrogate the acceleration of these fields during the Zika and Covid-19 emergencies. In so doing we bring together insights on speed and acceleration from the fields of sociology, science and technology studies (STS), and global health literatures. The concept of acceleration, particularly in the context of time, speed, and technological advancement, has been a central theme in sociological inquiry. This phenomenon is increasingly evident in high-speed societies where innovation and knowledge production have notably accelerated (Rosa, 2003). Scholars such as Webster (2019) and Felt (2017a; 2017b) highlight that many academic and technological fields now operate in an accelerated mode, driven by the pursuit of research outputs, wealth, and biovalue (Gardner & Webster, 2017). This acceleration is not uniformly distributed; it is supported by a complex technical and institutional infrastructure that enables innovation in particular research fields (cf. Morrison, 2017; Stephens et al., 2021). Infrastructures thus benefit certain groups or actors; as Wajcman and Dodd (2016) note, where the “powerful are fast, the powerless are slow.” This uneven distribution reflects broader inequalities in the ability to mobilise speed as a resource.

In global health, acceleration of research and development (R&D) is often seen as a time-limited response to extraordinary events like the Ebola, Zika, and COVID-19 outbreaks (Jensen et al., 2023; Kelly, 2018; Kelly et al., 2020). These crises have prompted complex institutional and political arrangements, leading to what is termed the ‘paradigm of emergency R&D’ (Kelly et al., 2022). The World Health Organization (WHO) has played a pivotal role in this paradigm, emphasising R&D as a core component of the outbreak response. The WHO's ‘R&D Blueprint’ and other policy documents (WHO, 2020a, 2020b, 2016a, 2016b, n.d.) underscore the importance of rapid research activation during epidemics, positioning it as essential to both immediate and future outbreak preparedness. This approach is framed as a moral imperative, advocating for collaboration, solidarity, and equitable access to innovations developed through accelerated research efforts.

## Methods

### Analytical approach: exploring the social and ethical dimensions of organoid technologies

The main research question animating our study is: How did the acceleration of organoid research during the Zika and Covid-19 emergencies impact the sustainability and focus of this research, and what social and ethical implications arise from these dynamics? This question emerged from our previous multi-sited ethnography of organoid technology that we conducted in several laboratories and locations in Global North countries (Bea & Hinterberger, 2025; Hinterberger & Bea, 2023). At the core of our ethnography – and at the core of the research of the scientists we interacted with – were often common complex diseases, such as diabetes, neurological disorders, cancers and gut and kidney conditions. Over the years and through a series of visits in labs and interviews with scientists, we learned how much effort and resources were going into developing complex 3D cellular cultures to study diseases prevalent in high-income countries. By contrast, there were fewer discussions or lab projects on how organoid research could advance studies of infectious diseases.

This changed dramatically during the Covid-19 pandemic, which brought our fieldwork to a stop and altered the course of some organoid research. Although away from the laboratory, we followed – through online interviews, academic pre-prints and media reports – the developments in the organoid field which, as key scientists noted, ‘went viral’ (Clevers, 2020) during the pandemic, now focusing predominantly on understanding the novel coronavirus. Such emergency acceleration of organoid research for infectious diseases, though remarkable, was not entirely new. Several years earlier, the field witnessed a similar ‘explosion’ (Yan et al., 2024) of infectious disease research prompted by the Zika virus outbreak in the Americas in 2015-2016.

Our study offers a sociological analysis of how organoid research for infectious diseases accelerated during the Zika and Covid-19 emergencies. We pay particular attention to the temporalities of research intensification, as well as to the practice and the discourses of acceleration. In this way we seek to provide a more nuanced account of acceleration by focusing on ‘non-linear rhythms of innovation’ (Webster, 2019, p. 4) and on the fluctuations of research rather than assuming an unproblematic rise of new technologies (cf. Wajcman & Dodd, 2016, p. 2). Exploring how accelerated research is being conducted and talked about provides the social context of acceleration and helps highlight potential tensions between the practice of acceleration and the discourses conveying normative assumptions and expectations about ‘how research and innovation (should) work’ (Felt, 2017b, p. 8; see also Webster, 2019).

### Sources and methodological considerations

Our analysis of the R&D acceleration in the field of organoids draws on a multi-sited ethnographic work and in-person and online interviews conducted with scientists working in this area of research. We supplemented our data with the analysis of secondary sources, such as popular media articles and grey literature. The analysis of these sources allowed us to situate the findings from our ethnography and to gain a better understanding of the discourses surrounding this research in the public realm.

**Figure 1.**
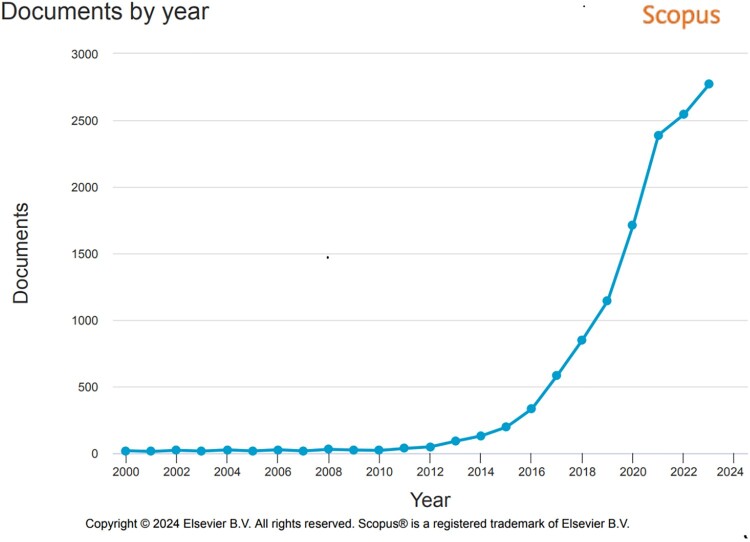
The rise of the organoid field in the years 2000-2023, according to the database Scopus (searches with a key word ‘organoids’). Research on organoid cultures, in the current sense of this term, has been emerging since about 2009. In that year, Scopus recorded only 23 scientific articles containing the word ‘organoids’ in the title, abstract or keywords**.** In 2023 alone, 2768 articles on organoids appeared in scientific journals.

**Figure 2.**
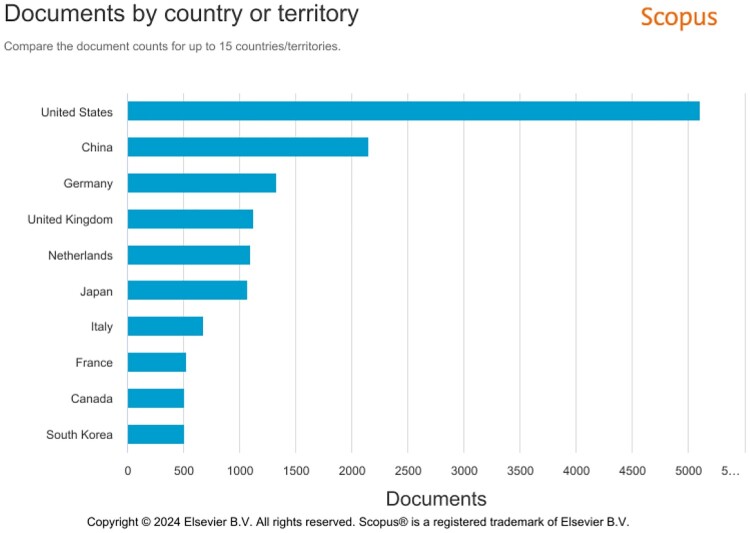
The geographical distribution of organoid research in the years 2000-2023, according to the database Scopus (searches with a key word ‘organoids’). The figure represents the volume of studies conducted in this field between 2009-2023. It shows that this research is concentrated in the countries of the Global North and/or with high scientific capabilities and infrastructures.

In the next step, we sought to follow on our initial observations about the acceleration of research in the organoid field, including an increase in the volume of published research. To this end, we conducted exploratory searches in the citation database Scopus. We confined our search to years 2000–2023, the period that saw the emergence of organoid research in the current sense of this term (cf. Corrò et al., 2020). We used a wide range of key words, such as ‘organoids’, ‘cerebral organoids’, ‘organoids’ AND ‘cancer’, ‘organoids’ AND ‘infectious disease’, ‘organoids’ AND ‘virus’, and so on. These exploratory searches, though limited in scope, provided us with a better understanding of how this research developed. To illustrate general trends in this field, we provide Figure 1 which shows the overall rise of organoid studies over the last 15 years, and Figure 2 which maps the geographical locations of key research centres. To triangulate our initial observations and our searches in Scopus, we drew on two types of secondary data: (i) we analysed scientific reviews of the organoid field which discussed progress of this research over time; and (ii) we examined bibliometric studies of the organoid field which used complex quantitative analyses to assess the dynamics of knowledge production in different subareas of the organoid field. Combining heterogeneous primary and secondary data allowed for complementarity and gaining insights into various aspects of the acceleration of organoid research.

## Results

### Acceleration of organoid research on the Zika virus

When in 2015 the Zika virus (ZIKV) outbreak started in Brazil, the virus had not previously featured on the epidemiological map of global health threats. Discovered in Uganda in the 1940s and transmitted by mosquitoes in tropical and subtropical regions of the world, Zika was believed to be rare and to cause only mild symptoms, such as a rash, fever and muscle pain. For a long time Zika did not attract much scientific attention, which meant that ‘during the first 60 years of its known existence, epidemic ZIKV was never reported’ (Gubler et al., 2017, p. S860).

This started to change at the turn of the millennium, when the Zika virus first caused a significant outbreak in Micronesia in 2007, and then in 2013 in French Polynesia, reaching Brazil around 2013-2014, and from there spreading to neighbouring countries (Gubler et al., 2017). The epidemiological maps of the spread of Zika published at that time by the US Centre for Disease Control and Prevention (CDC), marked vast parts of the Americas as areas of active virus transmission (Hennessey et al., 2016), with the potential of spilling beyond the mosquito-endemic regions as well. The virus, as the WHO estimated, was expected to infect 3–4 million people in the next twelve months, and, as Dr Margaret Chan, Director General of the WHO put it, it was ‘now spreading explosively in the Americas’ (Botelho, 2016), causing global health alarm. In Brazil, the virus was linked to over 4,000 cases of neonatal microcephaly, a severe and usually rare brain malformation resulting in a reduced size of the newborn's head and impaired motor, visual and cognitive functions. Although the link between Zika infection in pregnancy and congenital microcephaly in newborns was strong, scientists had no proof of causality and little was known about the virus, including whether it could be transmitted sexually, or vertically between the pregnant woman and her baby (Gyawali et al., 2016).

In the absence of reliable evidence about how the pathogen behaved, and with no diagnostic tools allowing for a fast detection, the Zika virus became the catalyst of a public health crisis. It also became a catalyst of ‘emergency R&D’ activities, when in February 2016 the WHO declared Zika a public health emergency of international concern (PHEIC) (Kelly et al., 2020). The international stem cell research community came to play a key part in this emergency response by developing organoids, miniature-like organs that modelled Zika infection in the human body. The research acceleration that ensued involved research groups based in Brazil, the US, China, and other high-income countries. As one of the research teams involved in this research recounted,

We urgently needed the answer to this question, given its public health implications. (…) All three of us (Guo-li, Hongjun, and Hengli) felt an increasing sense of urgency when the WHO announced the PHEIC regarding ZIKV (Ming et al., 2017, p. 750).

Organoid studies conducted soon after and at a record speed confirmed the link between the virus and neurological disorders in newborns. These discoveries contributed to the CDC's decision to announce in April 2016 that Zika did indeed cause microcephaly in newborns (Ming et al., 2017).

This achievement was even more remarkable, given that organoid technologies had not been on the radar of the global health community at that time and not featured on the WHO Zika Virus Research Agenda (WHO, 2016b). As the Zika emergency unfolded in Brazil, organoid technologies, though not widely known or available at the time, offered a way of studying how the pathogen could potentially be affecting the brains of babies growing in the womb. Only a few years earlier, scientists had created three-dimensional cerebral organoids out of human iPSCs which provided a model of early brain development and microcephaly.

Drawing on these previous studies, numerous international research teams started growing 3D miniature-like brain structures out of human stem cells and infecting them with the Zika virus. This novel approach presented unique advantages, as compared to traditional animal studies. As Marrazzo and colleagues (2021), have argued ‘Whenever a new viral threat emerges, rodents are often the most common animal model taken in consideration but not necessarily a guarantee of a proper human surrogate’ (p. 8). This was also the case with the Zika virus, which was shown to infect mice; it was not clear, however, whether it was able to affect animals in the same ways as humans (Vogel, 2017). Given the differences in the structure of the human and rodent brain, organoids proved a much better tool than the usual animal models in uncovering the impact of the Zika virus on the developing human brain. While infected fetal tissues were not available for research, brain organoids could mimic much of the structure of the developing fetal brain and trace the impact of the virus on the fetal brain in the first, second and third trimester of pregnancy. Organoids were thus capable of overcoming the shortcomings of both 2D cultures as well as animal models in a situation, where reliable knowledge about new infectious vectors was needed urgently (Marrazzo et al., 2021).

Just weeks after the WHO declaration of emergency, the first organoid studies on Zika were published in the spring of 2016 (Garcez et al., 2016; Tang et al., 2016), providing the first model of Zika infection and showing the damage the virus wrought on the developing brain. Another organoid study (Cugola et al., 2016) demonstrated that the Brazilian strain of the Zika virus was more dangerous than the original African one and capable of attacking the fetal brain. A range of other organoid studies appeared the same year bringing novel insights into the pathogenesis of the virus. The studies showed that while infection with Zika was associated with fetal anomalies during all the trimesters of pregnancy, the highest risk occurred during the first and second trimester, even among pregnant women without clear symptoms of Zika infection. In addition, while microcephaly and miscarriage attracted the most attention, other consequences of infection *in utero* were uncovered, including blindness and neurocognitive abnormalities (Majolo et al., 2019). Scientists also used organoids to screen chemical compounds to identify those that could be used to develop drugs for the Zika virus (Xu et al., 2016).

The widespread alarm caused by Zika meant that organoid research was closely followed by the media around the world. Cerebral organoids, or ‘tiny brains’ were portrayed as ground-breaking technologies helping fight new epidemics, while scientists at the forefront of this research appeared in high profile newspapers and media stations. In the public realm, celebratory discourses about research acceleration painted the picture of concerted scientific efforts, bolstered by the language and imagery of unfolding military operations to combat Zika (Löwy, 2024). They obscured, however, the spontaneous and unpredicted character of scientific collaborations. Many scientists involved in the emergency R&D for Zika found themselves working across various fields, often outside of their specialism, and fighting various challenges, such as shortages of samples, lack of equipment and gaps in expertise. To continue research, scientists relied on speedy identification of new potential collaborators, exploitation of existing networks, impromptu meetings, and even family members enrolled, in one prominent case, to optimise the work of laboratory equipment (Ming et al., 2017).

When the Zika emergency was declared in February 2016, the organoid field was novel and focused mostly on the study of development and common complex diseases, and not infectious ones. Then, within the space of a few weeks, which, as *The New York Times* noted, are ‘a nanosecond compared with typical scientific research time’, organoid technologies were able to address the key scientific gap of how the Zika virus could cause microcephaly in babies (Belluck, 2016). When in November 2016 the WHO announced the end of the Zika virus emergency, the newly emerging field of organoid research for infectious diseases was credited with an outstanding contribution to the outbreak response. According to commentators, ‘Human organoids uniquely helped researchers in the world collective fight against Zika disease’ (Marrazzo et al., 2021, p. 13), and were held as a proof that organoids could and should be used in research for emergent diseases posing global health threat.

### Acceleration of organoid research on COVID-19

Global health emergencies, as Kelly (2018) notes, rely on the ‘imaginary of unprecedented events’, generating collective distress and provoking action to address uncertainty about emerging pathogens. When in January 2020 the WHO declared the emerging coronavirus disease as a public health emergency of international concern, and in March described the outbreak as a pandemic, this emergency, too, felt unprecedented, especially given ‘the geographic trajectory of the SARS-COV-2 virus, with rates of infection rising explosively in high-income countries’ (Kelly et al., 2022, p. 200).

In response, in March 2020 the WHO published its coordinated global research roadmap. The document set out research priorities and aimed at accelerating and coordinating efforts to understand the novel pathogen, and develop vaccines, tests, and treatments. ‘The global imperative for the research community,’ the roadmap stated, ‘is to maintain a high-level discussion platform which enables consensus on strategic directions, nurtures scientific collaborations, and supports optimal and rapid research to address crucial gaps’ (WHO, 2020b). In contrast to the emergency R&D roadmap for the Zika virus, this time the emergency R&D plan for Covid-19 included organoid technologies, listed as one of key research priorities crucial for understanding the behaviour of the new virus. In practice, the field of organoids contributed to many more research areas and key priorities, such as for example drug screening and providing insights about subsequent coronavirus variants that emerged throughout the pandemic. Indeed, as popular media put it, organoids constituted not just a technology, but ‘a possible weapon against the pandemic’ (Rosen, 2020).

The declaration of the pandemic led to a scientific mobilisation of the global research community (Srivastava et al., 2020). The stem cell and organoid research community mobilised early on during the pandemic. Academic journals and research centres promoted networking events, such as online meetings and seminars, and encouraged knowledge and data sharing as well as publishing pre-prints (Mummery, 2020). The initial shock of the pandemic and lockdowns quickly gave way to a sense of urgency. As one of the scientists we interviewed recalled:

It was such a particular moment, and the fact that everybody got together, and they dropped everything else that they were doing [and that] everybody was working on Covid. (…) it felt good to be a part of something of a national emergency, of a global emergency and that we could contribute somehow. (Interview Participant)

This acceleration of research resulted in a ‘huge and sudden increase in publications about organoids as effective in vitro models for unraveling host-viral interactions’ (Marrazzo et al., 2021, p. 13). Organoid research proved vital for elucidating some of the key scientific questions throughout the pandemic. When, at the start of the outbreak, there was uncertainty about the potential sources of the novel pathogen, organoid studies provided experimental evidence that coronavirus could come from bats, a first study of its kind that was celebrated in an article published in *Nature* (Mallapaty, 2021).

Organoids, especially those derived from human cells, were quickly and widely adopted for studying the novel coronavirus. The technology was versatile and with clear advantages to animal models. This is because, as scientists soon discovered, coronavirus affected humans in ways that were different to laboratory animals used in disease modelling (Corsini & Knoblich, 2022). For example, mice turned out not to be susceptible to infection with wild-type SARS-CoV-2 virus, while ferrets displayed only mild symptoms, mainly in upper respiratory tract (van der Vaart et al., 2021).

Human organoids, by contrast, had no such limitations, and could be swiftly developed to answer research questions. As SARS-CoV-2 affects multiple organs and systems, a wide range of organoids were developed to study the ways in which the virus enters the cells, and how it affects the human body, including the upper respiratory tract and lungs, brain, kidney, liver, heart, blood vessels and intestine (Han et al., 2022). Organoid studies helped understand how the virus could lead to a wide range of symptoms, from pneumonia, through to diarrhea, hearing loss or anosmia (Corsini & Knoblich, 2022), and how it could trigger an immune reaction called a cytokine storm that could be fatal to the infected person (Han et al., 2022). In addition, organoids were used in drug screening to select effective Covid-19 treatments, including antivirals. Further studies of subsequent coronavirus variants followed, demonstrating for example that the Alpha variant of coronavirus was much more transmissible than the original strain, and that the Omicron variant was less successful at infecting lungs (Kim et al., 2022). The ability of organoids to be applied to a wide range of research questions about the novel pathogen led to an ‘exponential adoption’ of this technology and significant contribution to the pandemic response (Tran et al., 2022, p. 16).

International collaborations were key to the success of this organoid research, and they relied on division of labour and expertise. Organoid researchers often lacked expertise or facilities, such as high biosecurity laboratories, to work with emerging pathogens. Virologists, on the other hand, had the facilities and even samples of the virus at hand, but did not have the necessary skills and experience to grow viable organoids. Collaborations were therefore essential, and often started via informal channels, for example by calling a friend or re-connecting with colleagues from other institutions. As one of the scientists recounted in an interview:

It all started at a conference a year before [where we met our future collaborators]. So when the pandemic started, they approached us and asked if we would like to work with them on the coronavirus. We had organoids developed in our lab and were ready to share them with the other team for experiments (Interview Participant).

For this participant, the Covid-19 collaboration continued over an extended period and was very intensive, filling all the time and resources available for research. Although proud of their research group's scientific achievements, this participant also talked about exhaustion and acknowledged that the unprecedented pace of research acceleration was difficult to maintain in the long term.

Despite the challenges of research acceleration, the Covid-19 pandemic presented, as many commentators hoped, a turning point for organoid studies for infectious diseases. Numerous scientific reviews and commentaries on the progress of this field envisaged an expansion of research on infectious diseases to address global health challenges. This included organoid research for neglected diseases as well as development of organoid platforms for pandemic preparedness (Blutt & Estes, 2022; Han et al., 2022). Given the versatility of organoid technologies, the possibilities seemed endless. As one scientist explained in *Nature*: ‘These are magical cultures. It's just your imagination that limits where this field can go’ (Mallapaty, 2021).

### Between Zika and COVID-19: deceleration of organoid research

As we have shown, organoid technologies have emerged as a success story of R&D acceleration, making a unique and significant contribution to Zika and Covid-19 responses. The sudden rise of organoid research for infectious diseases has been reflected in celebratory discourses in the public realm. It has also been captured in bibliometric studies and review articles which traced developments in the organoid field (e.g. Shoji et al., 2024; Yan et al., 2024; Zhang et al., 2022). These studies suggest that the ‘explosions’, or peaks in scientific activities, were interspersed with periods when the number of published research dipped or plateaued, and that the hot topics of infectious disease studies were soon turning cold when outbreaks receded. To better understand the acceleration and deceleration of organoid research for infectious diseases, it is useful to scan the broader landscape of the organoid field, and we used data from Scopus and published bibliometric research to look at the patterns of knowledge production.

The field of organoid research is relatively recent, with significant developments in organoid cultures emerging only in the past fifteen years or so (Figure 1) (for a historical overview of this field see for example Corrò et al., 2020).

From about 2011, the organoid field experienced remarkable growth, which was attributed to the enormous potential of organoids and their enthusiastic uptake in diverse areas of research (Zhang et al., 2022). However, even though the volume of organoid research has been expanding rapidly, the numbers are still relatively low, especially in comparison to other fields of the life sciences. This is because organoids are still an emerging field and significant challenges exist to move this research forward: growing organoids requires specific expertise and skill sets, is expensive and labour intensive (Bea & Hinterberger, 2025). It is therefore not surprising to see that, currently, organoid research is concentrated in high-income Global North countries, such as the US, Germany, the UK, and the Netherlands, with China also a key player in this field (Figure 2). According to bibliometric studies, research conducted in the US, China, Germany, Japan and the Netherlands accounts for 94 percent of all research outputs in this field, with high volume of interconnected and collaborative networks between scientists based in these countries (Li et al., 2024).

From the very beginning, the focus of the field was on studies of development, disease modelling, and, increasingly, precision medicine and drug screening (Zhang et al., 2022). Much of this research has concentrated on common complex diseases, for example neurological and brain disorders such as Alzheimer's, as well as cancer (Shoji et al., 2024). Studies on cancer modelling with the use of organoids are generously supported by international funders. For example, a collaboration between the US National Institute of Health, Cancer Research UK, the Wellcome Sanger Institute, and the Dutch foundation Hubrecht Organoid Technology has led to the establishment of the Human Cancer Models Initiative, with the aim of developing up to 1,000 next-generation cancer models that could be shared and used by researchers around the world (National Cancer Institute, n.d.). Organoid Cell Atlas is another well-funded initiative aimed at ‘accelerating disease-centric research in areas such as rare genetic diseases, complex multifactorial diseases and precision oncology’ (Bock et al., 2021, p. 16).

By contrast, there has been no comparable initiative in infectious disease research. Since the inception of the field, the uptake of organoid technologies was much slower for infectious disease research, even though breakthrough early studies on such infections as norovirus showed a great potential of organoid technologies in this area (Tran et al., 2022).

The Zika emergency marked an important moment for organoid research, showcasing its capabilities in addressing urgent questions in global health, at speed and in conditions of high uncertainty. The scientific acceleration prompted by the emergency was concentrated in countries most affected by the outbreak, and with the highest research capacity and available funding, such as Brazil, the US, and China (Oliveira et al., 2020; Shoji et al., 2024). However, as the Zika outbreak receded, funding for this research decreased (Head et al., 2020). After the surge of interest in organoid research on Zika in 2016, the number of publications in this area started falling in 2017 and 2018 (Majolo et al., 2019). This happened despite significant gaps remaining in the understanding of the long-term consequences of Zika infection for the affected children and uncertainty surrounding their clinical management (Majolo et al., 2019). While in the aftermath of the outbreak some of the scientific papers on organoids and Zika remained in the top ten of all citations of organoid research (Zhang et al., 2022), the area of infectious disease research slipped off the radar again. Until the advent of the Covid-19 pandemic it remained largely overlooked (Tran et al., 2022), except for studies on influenza and respiratory syncytial virus (Geurts et al., 2021).

The emergence of the novel coronavirus in 2020 marked another shift for the organoid field, resulting in a widespread adoption of organoid technologies for studies of the virus in Global North countries (Tran et al., 2022). However, what looked like an ‘explosion’ (Yan et al., 2024) in organoid research was a rapid uptake of the technology from a relatively low level. In the run-up to the pandemic, as Vincan (2023) noted, the potential of this research was not realised. During that period

[r]esearchers have developed organoids from a vast array of organs, including the gut, stomach, liver, brain, and kidneys, to understand how tissues develop and repair. However, the potential of these organoids as authentic infection models was largely not embraced until the COVID-19 pandemic [5]. COVID-19 has been described as a “rampage through the body” [8], and organoids established from diverse organs were adopted globally in the scramble to develop therapies to combat COVID-19. (Vincan, 2023, p. 120)

The pandemic prompted an acceleration of development of many types of organoids to model the behaviour of the coronavirus in the human body, but it was especially marked in the case of lung organoids. This area of organoid research was underdeveloped prior to the pandemic, even though respiratory conditions constitute a leading cause of death across the world (Shoji et al., 2024).

The contribution of organoid technologies to the Covid-19 response has fueled hopes that infectious disease studies will now gain greater prominence in various areas of global health research, such as pandemic preparedness and emerging pathogens, neglected tropical diseases, as well as antimicrobial resistance or HIV (Blutt & Estes, 2022; Geurts et al., 2021; Marrazzo et al., 2021). The acceleration of organoid research during the pandemic even prompted some commentators to declare that ‘the era of organoids modelling infectious disease has arrived’ (Tran et al., 2022, p. 3). It remains to be seen, however, if these hopes will materialise. As one of the scientists we interviewed observed: ‘I don't think there has been anything done with organoids concerning tuberculosis, leprosy, and other diseases of the developing world. (…) In the past four years nothing happened, but there are some initiatives here’. However, this participant's hopes for more research in this area were mixed with doubts. This research agenda did not seem to carry ‘a very big paper appeal for the global community’, and in this participant's view funders could be more inclined to support research on pandemic preparedness, as ‘those are threats to everybody, in every country’.

The researchers we interviewed noted that the ‘R&D’ acceleration during Zika and Covid-19 emergency was unprecedented and relied on the limited resources and intensive labour of the organoid research community. Without ‘very significant investment’, they argued, research on infectious diseases and pandemic preparedness would not be possible. As one participant recalled: ‘It was crazy, people putting a lot of effort in it in a very short time – but this cannot be maintained. (…) We cannot do this for everything’.

Access to funding remained a key issue as infectious disease research did not seem to be a priority in funding calls. A scientist we spoke to recounted how in the run-up to the pandemic their research proposal on infectious diseases was turned down for funding, only to be awarded many months later, when the novel coronavirus emerged. The unpredictability of funding for infectious disease research contrasted with the availability of funds for studying chronic diseases. In a popular article on ‘How to stop the next pandemic’, a prominent organoid expert expressed frustration about the mindset of funders and investors in the run-up to Covid-19 emergency. Discussing the potential of organoids for tackling pandemics this scientist claimed that:

This technology has been available, but nobody really cared. (…) A year ago, I would go to investors and tell them, ‘We have this cool new way to study viruses which could lead to new drug targets,’ and I was basically shown out of the office. Instead, they were investing in their 120th company on cancer. We all knew there might be a pandemic, but nobody believed it. (Cox, 2021)

As the Covid-19 emergency ended, the scientists we interviewed expressed hopes that organoid studies on the SARS-CoV-2 would continue, and that new methods and platforms would be developed to advance research in infectious diseases in general (Chau & Sugimura, 2024).

## Uneven trajectories in organoid research: insights into acceleration, deceleration, and ethical implications

Above we have sought to understand the temporalities of the acceleration of organoid research during emergencies and at other times. We have shown that from their inception, organoid technologies have been developed in high-income countries of the Global North and in countries with high scientific capabilities, such as China. Scientific advances in this field have reflected Global North research priorities and interests with common complex diseases such as neurological and brain conditions and cancer. Research in these areas has been well supported and indeed it has enjoyed an upward growth trajectory from about 2009 onwards, a trend that attracted a lot of attention and hype (Huch et al., 2017).

By contrast, celebratory discourses surrounding the acceleration of organoid research to combat Zika and Covid-19 emergencies disguise a patchy uptake of organoid studies to address the challenges of infectious disease. Organoid research in this area was turbo-charged during the Zika and Covid-19 emergencies, and it concentrated on a limited set of questions to better understand the new pathogens and to support the outbreak response. However, when emergencies ended, organoid research on infectious diseases decelerated and remained underdeveloped. In this case, rather than following a path of a steady rise, acceleration followed a ‘non-linear rhythm of innovation’ (Webster, 2019) and consisted of time-limited ‘explosions’ (Yan et al., 2024), or outbursts of research activities, which slowed down when research funding ended, and the threats of outbreaks decreased. Meanwhile, the main focus of the organoid field remained on addressing common complex diseases prevalent in the Global North, and research in this area kept progressing. The different trajectories of these technologies in different research areas reflect the inequities in global health where speed of research is available to those with power and resources (Wajcman & Dodd, 2016), and where the Global North countries tend to prioritise what they consider their own needs over those of the Global South.

### The ethics of speeding up and slowing down

Our study reveals tensions between the discourses and the practice of research acceleration during health emergencies. The WHO policy documents outlining the principles of emergency R&D focus predominantly on ethical aspects of acceleration, highlighting the notion of the common good, mobilisation, solidarity, collaboration, openness and sharing of the results. But this understanding shifts attention away from the issue of infrastructures that are key for the acceleration to take place. Our analysis found that ethical principles of the mobilisation for the common good did indeed underpin many activities of organoid researchers who took part in the emergency response. The ideas of collaboration underpinned scientists’ attempt to temporarily plug the gaps in existing research infrastructures by sharing resources such as lab equipment, samples, data, and expertise, and by working overtime in areas often outside of their specialism. While these efforts, fueled by emergency funding, did lead to scientific breakthroughs, they were not sustainable in the longer terms, when in the aftermath of the outbreak the funds for infectious disease response were diverted elsewhere.

The existing infrastructures governing research and priority setting in the Global North are thus in conflict with the notion of the ‘common good’ advanced by the WHO. This means that ‘the moral obligation to learn as much as possible as quickly as possible’ (WHO, 2016a) is fulfilled only when public health challenges turn global, thus becoming everyone's problem. This contradicts the assumption that the ‘emergency R&D’ should help tackle the ongoing outbreaks and prepare for the future ones. In the case of organoid research for infectious diseases, the current model of emergency R&D did not lead to sustained efforts to enhance preparedness, and it focused on tackling short-term issues.

Overall, the patterns of acceleration and deceleration in organoid research reflect broader issues of equity, fairness, and responsibility in global health and biomedical research. Our study reveals that while organoid technologies significantly advanced during the Zika and COVID-19 emergencies, their development experienced a notable deceleration post-crisis, reflecting broader issues of equity in global health research. Despite the initial surge in research driven by urgent health needs, organoid studies predominantly addressed diseases prevalent in high-income countries, leaving gaps in the exploration of infectious diseases. This shift exemplifies the challenges of ‘emergency R&D’ and highlights the need for a more sustained and equitable approach to research priorities. Our findings underscore the ethical implications of these dynamics, including the disparities in research focus and funding, and call for an expanded ethical analysis that includes global health challenges beyond the immediate crisis. For example, Parker et al. (2024) emphasise the need for a broader understanding and practice of ethics during and beyond health emergencies. Drawing on London (2021), they argue that ‘research is not a morally optional activity in a global health emergency’ and that policymakers, academics, research funders and health systems have the responsibility to generate knowledge both during health emergencies and beyond ‘to inform responses to future emergencies and provide an evidence base for future generations’ (Parker et al., 2024, p. 5).

In this context, we argue that the lack of institutional and material infrastructures to support organoid research for infectious diseases presents an ethical problem. In her recent International Association of Bioethics Presidential Address, Nancy Jecker (Jecker, 2024) draws attention to the assumption prevailing until recently that infectious diseases had been conquered and therefore did not feature ‘front and center’ (p. 5) in the bioethics research agenda. This has resulted in a skewed focus on individual autonomy and informed consent in the context of bioethics as it has developed in North America. Our findings about the trajectory of organoid research are consistent with this argument, and they show that infectious disease studies were not heavily prioritised or funded in research agendas.

Along with this, the majority of ethical analyses of organoid technologies continue to focus on the moral status of organoids, especially brain organoids, in relation to the human body (e.g. Jeziorski et al., 2023). However, this can perpetuate a narrow set of bioethical concerns that tend to downplay the global burden of infectious disease and the concerns of global health research. Our findings indicate that current social and ethical analysis of organoid technologies tends to overlook the prioritisation of common complex diseases at the expense of infectious diseases, and they suggest a need to expand the focus of bioethics to the issues of global burden of infectious disease and research infrastructures.

Furthermore, the relationship between the acceleration of organoid research and the status of animal models in research reveals evolving transformations that are part of new ethical terrains. The use of organoids has the potential to reduce reliance on animal models, which has been a significant issue in drug development and ethical debates (Hinterberger & Bea, 2023). However, shifts toward organoid research do not entirely replace traditional animal models but rather have introduced new forms of knowledge production that are influenced by the global and market-driven forces. The ‘explosion’ of organoid research during emergencies and its subsequent deceleration raises questions about the broader implications of acceleration in research. It prompts us to consider whether acceleration is merely a reflection of increased funding and publication rates or if it represents a fundamental shift in research priorities and methodologies. Moreover, this transition underscores the ethical concerns of research prioritization, particularly in balancing immediate responses with long-term global health needs.

### Integrating bioengineering into global health frameworks

Ultimately, our findings suggest that the ethical and practical challenges of organoid research are intertwined with broader issues of research equity and sustainability. Addressing these challenges requires a more nuanced understanding of how bioengineering technologies fit within global health frameworks and how they can be effectively integrated into a research agenda that aligns with both immediate and future health priorities. As Street and colleagues have recently argued, there is a need to acknowledge the unequal conditions shaping global health research, ‘including questions about who funds, produces and distributes knowledge’, as well as the need to establish more equitable relationships within the innovation process’ (Street et al., 2024, pp. 445–446). This would require involving scientists and bioengineers from low- and middle-income countries in research and innovation processes. Such initiatives are starting to take place; for example the ISIDORe consortium funded by the EU unites advanced research infrastructures for studies on current and future infectious disease outbreaks, and it supports projects and collaborations with researchers from low- and middle-income countries (see ISIDORe, n.d.). In addition, the recent WHO's (2023) report ‘Emerging technologies and scientific innovations: a global public health perspective’ has identified organoids as some of the key technologies that could significantly impact global health research in future.

Our paper helps to create a better empirical picture of bioengineering in the context of global health. We need however further studies that explore these relationships. The disparity between the focus on common complex diseases prevalent in high-income countries versus infectious diseases affecting lower-income regions illustrates the uneven distribution of research priorities and resources. This reflects the structural inequalities in the production and deployment of global health knowledge that are highlighted by recent calls to decolonise global health by engineering equitable relationships (Editorial, 2024). By demonstrating that the acceleration of organoid research did not lead to a proportional increase in addressing global health needs, our paper challenges the assumption that increasing the accessibility of biomedical technologies inherently leads to global health equity.

## Conclusion

This study set out to examine social and ethical issues arising from organoid technologies in global health. We focused on the emergence of organoid technologies and the acceleration of this research for infectious disease studies, particularly as part of the response to health emergencies of Zika and Covid-19. We interrogated the accelerated mode of this research by exploring the temporalities of acceleration and its fluctuation over time. We also explored the discourses of acceleration in policy documents, grey literature and media accounts and the practice of acceleration of this research as recounted by life scientists at the forefront of this research. In our study we revealed the tension between ethical principles of acceleration of ‘emergency R&D’ and the practice of this research, including gaps in funding and infrastructures that have led to deceleration of organoid studies for infectious diseases after emergencies ended.

We have shown that organoid technologies played an important part in both emergencies and were used to model the behaviour of the novel pathogens and answer a wide range of research questions. In the case of the Zika virus outbreak, the acceleration of research on brain organoids helped address a key scientific gap and confirmed the link between Zika infection and neonatal microcephaly. In the case of the Covid-19 pandemic, new organoid models were rapidly developed to study the pathogenesis of the coronavirus and to screen drugs for treatment. In both cases, this led to significant discoveries as organoid technologies provided key insights into the workings of the novel pathogens that could not have been achieved with traditional animal models. These achievements were made possible by a mobilisation of research during global health emergencies.

Bioengineering is poised to play an increasingly prominent role in global health research (Bowden et al., 2023). Since the pandemic, there is a growing awareness among governments, funders, thinktanks and philanthropists of the role that bioengineering can play in pandemic preparedness and tackling infectious disease outbreaks, as well as an acknowledgment of the need to fund R&D for global health challenges (e.g. Policy Cures Research, n.d.; Stuart, 2021; UK Government, 2023; Wellcome Trust, 2023). In this changing landscape, organoids technologies have demonstrated their usefulness as a vital tool to study novel pathogens and ‘proved their value as an experimental virology platform’ (Clevers, 2020, p. 355). In this context, there are hopes that the potential of organoid technologies can finally be utilised to address global health challenges, especially for pandemic preparedness and research on neglected diseases. For this vision to materialise urgent changes are needed in the prioritisation and sustained funding of global health research agendas (Lurie et al., 2021; Yegros-Yegros et al., 2020). Otherwise, organoid technologies could be caught up again in the ‘politics of attention and neglect in global health’ (Jensen et al., 2021), and their potential for advancing infectious diseases research not realised.
